# Once in a Bile — the Incidence of Bile Reflux Post-Bariatric Surgery

**DOI:** 10.1007/s11695-022-05977-2

**Published:** 2022-02-28

**Authors:** Thomas A. Eldredge, Madison Bills, Ying Yang Ting, Mikayla Dimitri, Matthew M. Watson, Mark C. Harris, Jennifer C. Myers, Dylan L. Bartholomeusz, George K. Kiroff, Jonathan Shenfine

**Affiliations:** 1 Discipline of Surgery, University of Adelaide, Adelaide, South Australia Australia; 2 Department of Surgery, The Queen Elizabeth Hospital, 28 Woodville Road, Woodville South, Adelaide, South Australia 5011 Australia; 3 Department of Nuclear Medicine, PET and Bone Densitometry, Royal Adelaide Hospital and SA Medical Imaging, Adelaide, South Australia Australia; 4 Department of Gastroenterology, Royal Adelaide Hospital, Adelaide, South Australia Australia

**Keywords:** Bile reflux, One anastomosis gastric bypass, Sleeve gastrectomy, Roux-en-Y gastric bypass

## Abstract

**Purpose:**

Excellent metabolic improvement following one anastomosis gastric bypass (OAGB) remains compromised by the risk of esophageal bile reflux and theoretical carcinogenic potential. No ‘gold standard’ investigation exists for esophageal bile reflux, with diverse methods employed in the few studies evaluating it post-obesity surgery. As such, data on the incidence and severity of esophageal bile reflux is limited, with comparative studies lacking. This study aims to use specifically tailored biliary scintigraphy and upper gastrointestinal endoscopy protocols to evaluate esophageal bile reflux after OAGB, sleeve gastrectomy (SG) and Roux-en-Y gastric bypass (RYGB).

**Methods:**

Fifty-eight participants underwent OAGB (20), SG (15) or RYGB (23) between November 2018 and July 2020. Pre-operative reflux symptom assessment and gastroscopy were performed and repeated post-operatively at 6 months along with biliary scintigraphy.

**Results:**

Gastric reflux of bile was identified by biliary scintigraphy in 14 OAGB (70%), one RYGB (5%) and four SG participants (31%), with a mean of 2.9% (SD 1.5) reflux (% of total radioactivity). One participant (OAGB) demonstrated esophageal bile reflux. De novo macro- or microscopic gastroesophagitis occurred in 11 OAGB (58%), 8 SG (57%) and 7 RYGB (30%) participants. Thirteen participants had worsened reflux symptoms post-operatively (OAGB, 4; SG, 7; RYGB, 2). Scintigraphic esophageal bile reflux bore no statistical association with de novo gastroesophagitis or reflux symptoms.

**Conclusion:**

Despite high incidence of gastric bile reflux post-OAGB, esophageal bile reflux is rare. With scarce literature of tumour development post-OAGB, frequent low-volume gastric bile reflux likely bears little clinical consequence; however, longer-term studies are needed.

**Clinical Trial Registry:**

Australian New Zealand Clinical Trials Registry number ACTRN12618000806268.

**Graphical abstract:**

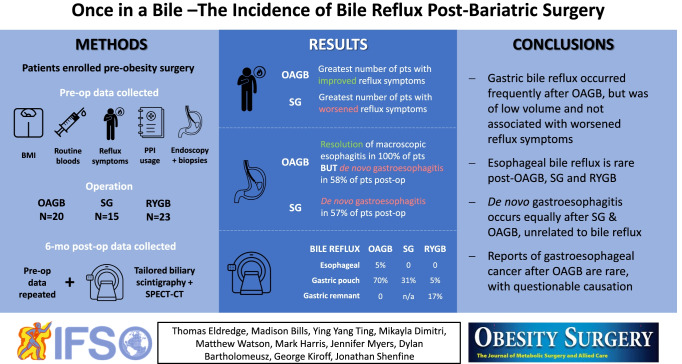

## Introduction

The one anastomosis gastric bypass (OAGB) is deservedly the third-most performed metabolic and obesity surgery (MOS) procedure globally [1]. Large meta-analyses demonstrate equivalent, if not superior, metabolic outcomes compared with Roux-en-Y gastric bypass (RYGB) and sleeve gastrectomy (SG), the two most popular MOS operations [2, 3]. Despite equivalence, OAGB has not been adopted with enthusiasm, due to concerns around the potential impact of post-operative esophageal bile reflux. This is often termed duodenogastroesophageal reflux (DGER), but strictly, this is not a correct term regarding post-OAGB anatomy. The potential for bile reflux stems from the anatomical similarities with both the Mason gastric bypass and the Billroth II procedure, both of which were also controversially associated with both duodenogastric and esophageal bile reflux and thus a potential cancer risk. No gold standard investigation exists to diagnose esophageal bile reflux, and heterogenous diagnostic protocols are evident in the few studies that evaluate esophageal bile reflux post-OAGB. This study aims to elucidate the incidence and severity of esophageal bile reflux post-OAGB, with direct comparison to SG and RYGB, by utilising a specifically developed diagnostic protocol tailored for a post-MOS cohort.

## Methods

### Participants

**Fig. 1:**
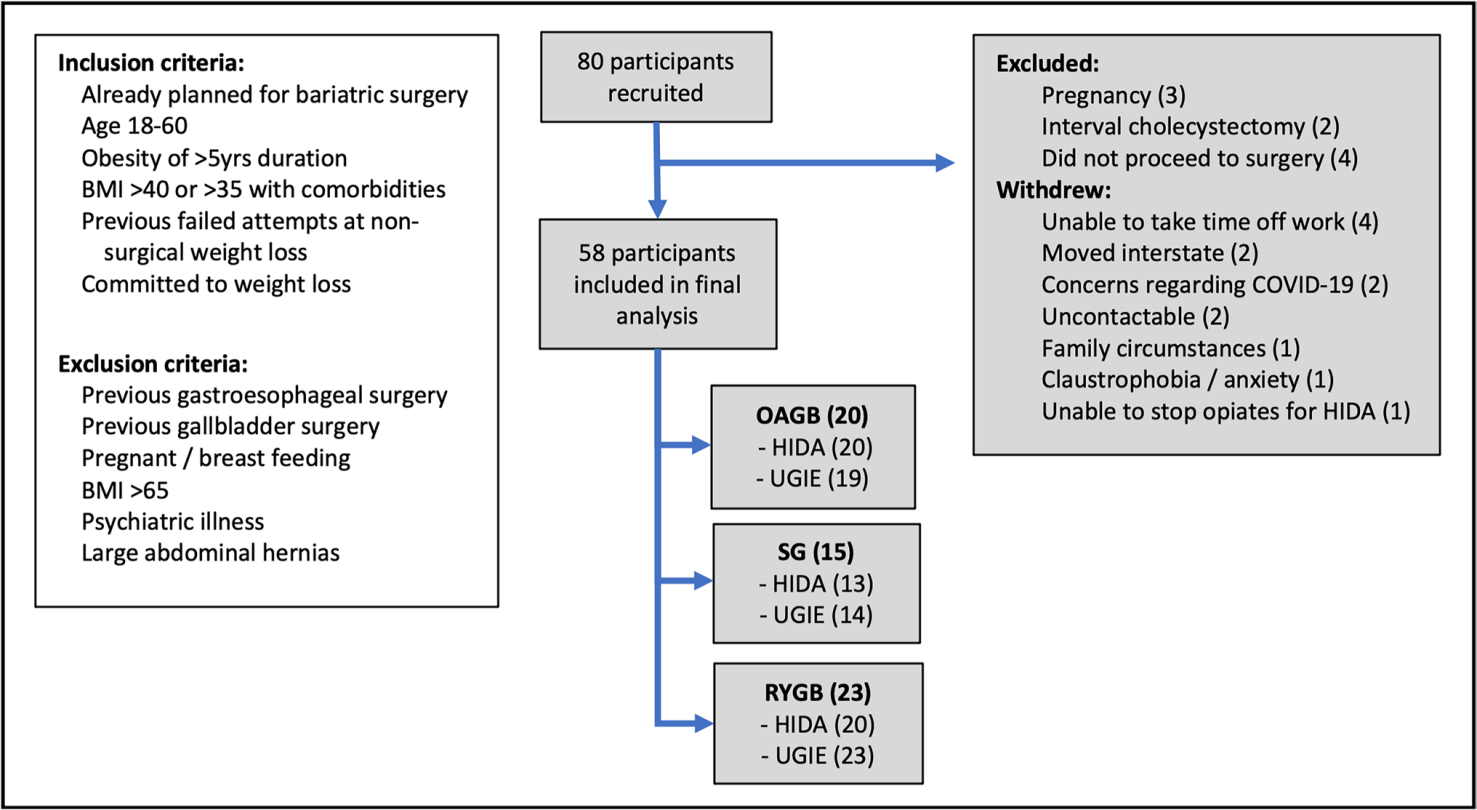
Participant inclusion flowchart. (BMI, body mass index; HIDA, hepatobiliary iminodiacetic acid scintigraphy; UGIE, upper gastrointestinal endoscopy)

Eligible participants (inclusion/exclusion criteria defined in Fig. 1) from multidisciplinary obesity clinics of two public and four private hospitals in Adelaide, Australia, were invited to participate. The type of procedure undertaken was determined by informed patient preference; participants undergoing OAGB, SG and RYGB were included, forming the three arms of this prospective cohort study. Randomisation to procedure was not possible due to lacking clinical equipoise.

To enable inferential statistical tests on study findings, a power analysis was conducted, which determined a target sample size of 24 participants per trial arm (72 total), using *Fisher’s exact conditional test for two proportions*, assuming a power of 80% and significance level or alpha of 0.05 (i.e. 80% or greater chance of finding a statistically significant difference when there is one). The calculation assumed a 45% difference between worst (50%) and best (5%) incidence of bile reflux between procedures, a ‘best estimate’ from limited published data. The calculation included design effect or variance inflation factor to account for multiple surgeons and an additional 10% of patients per arm (for potential loss to follow-up), to minimise bias related to attrition reducing the effective sample size.

Follow-up occurred at 6 months post-operatively, an expected timepoint at which participants have returned to a stable lifestyle. After study commencement, follow-up was impacted by government-mandated restrictions regarding elective medical investigations/treatments during the COVID-19 pandemic.

### Data Collection

The following data were collected pre-operatively and again at follow-up: full medical history; height, weight and body mass index (BMI); blood tests (lipid studies, Hba1c, fasting glucose, biochemical panel and full blood exam); reflux symptom assessment; and macro- and microscopic gastroesophageal assessment by upper gastrointestinal endoscopy (UGIE), along with gastric fluid aspiration for bilirubin analysis. In addition, tailored biliary scintigraphy was performed at 6 months post-operatively. No pre-operative scintigraphy was performed, an ethical decision to limit radiation exposure for participants.

### Symptom Assessment

The *GerdQ* was utilised as a validated, self-administered, patient-centred questionnaire with similar diagnostic accuracy for symptom-based diagnosis of gastroesophageal reflux to that of a gastroenterologist [4, 5].

### Endoscopy

Endoscopy was performed under intravenous sedation with oropharyngeal topical anaesthesia using lignocaine/phenylephrine (CoPhenylcaine™, ENT Technologies, Melbourne, Australia). Mucosal biopsies were taken from the gastric antrum (where present), gastro-jejunal anastomosis (where present), gastric body and distal oesophagus for histopathological analysis. The endoscopist documented the following: any visible bile in the stomach, macroscopic gastritis, esophagitis [6], anastomotic erosion/ulceration and details of any hiatus hernia.

Gastric fluid, aspirated via the endoscope channel, was immediately transferred to storage tubes and placed in a − 80 °C freezer prior to batched bilirubin analysis utilising enzymatic colorimetric assay [7] by an accredited state-wide pathology laboratory (SA Pathology, Adelaide, Australia).

### Biliary Scintigraphy

Our previously described modified biliary scintigraphy protocol, tailored for a post-MOS cohort [8], was performed 6 months post-operatively.

### Surgical Technique

Seven surgeons were involved in this study. Techniques were largely identical among surgeons, with differences outlined below. All procedures were completed laparoscopically or robotically (for 2 cases), with standard 4-port placement and an epigastric liver retractor. Stapled anastomoses utilised the EndoGIA™ or Signia™ stapling systems (Medtronic, Minneapolis, USA).

#### OAGB

One anastomosis gastric bypass was predominantly performed by one surgeon (*n* = 17/20). A stapled gastric pouch was fashioned over a 36 Fr. bougie, extending to the *incisura angularis*. Small variation in biliopancreatic (BP) limb length was observed (150–200 cm), and an antecolic, end-to-side gastro-jejunal stapled anastomosis was constructed. The remaining bowel defect was hand sewn with a 2/0 barbed, absorbable suture (V-loc™, Medtronic, Minneapolis, USA). An ‘anti-reflux’ stitch was placed between the afferent loop of the jejunum and the lateral aspect of the gastric pouch. Petersen’s space was not closed.

#### SG

Sleeve gastrectomy was performed by five surgeons. Two participants underwent robotic (da Vinci Xi, Intuitive Surgical, Sunnyvale, USA), rather than laparoscopic procedures, using identical port placement and a liver retraction device, and two participants had a concurrent cruroplasty. The stomach was mobilised along the greater curvature and posteriorly to visualise the diaphragmatic crura. Gastric stapling commenced ~ 6 cm proximal to the pylorus, ending at the angle of His with a 36 Fr. bougie (or 48 Fr./52 Fr. in 2 cases) for luminal calibration.

#### RYGB

Roux-en-Y gastric bypass was performed by five surgeons. Minor variations in BP- and alimentary limb lengths were observed. A small, stapled gastric pouch was fashioned over a 36 Fr. Bougie. An antecolic, end-to-side gastro-jejunal stapled anastomosis was performed with a 50–80 cm BP limb. A stapled jejuno-jejunostomy was performed using a 100–110 cm alimentary limb. The resulting anastomotic defect was closed with 2/0 monofilament (Monocryl®, Ethicon, Somerville, USA) or barbed V-loc™ absorbable suture. Mesenteric defects were routinely closed.

### Statistics

Statistical analysis was undertaken by the lead author (TE), with assistance of our unit’s statistician. Descriptive statistics included mean (standard deviation) or median (interquartile range) as appropriate binary and ordinal logistic generalized estimating equations models, and linear mixed-effect models were applied depending on the outcome variable (binary, ordinal or continuous), controlling for repeated measures over time and adjusting for clustering on hospitals. *p*-Values of < 0.05 were considered significant.

## Results

### Participants

Eighty participants were invited to participate between November 2018 and July 2020. Nine participants were excluded after enrolment and 13 withdrew; thus, 58 participants were evaluated before and after surgery: OAGB, *n* = 20; SG, *n* = 15; and RYGB, *n* = 23 (see Fig. 1). Forty-five participants were female (78%), and participant’s mean age was 41.8 years (SD 9.92). Mean time lapse from surgery to follow-up endoscopy was 250 days (SD 90.6), and time to biliary scintigraphy was 248 days (SD 65.5). Pandemic-related shutdowns resulted in a bimodal distribution of follow-up times, with peaks at 200 and 300 days.

### Reflux Symptoms

**Fig. 2:**
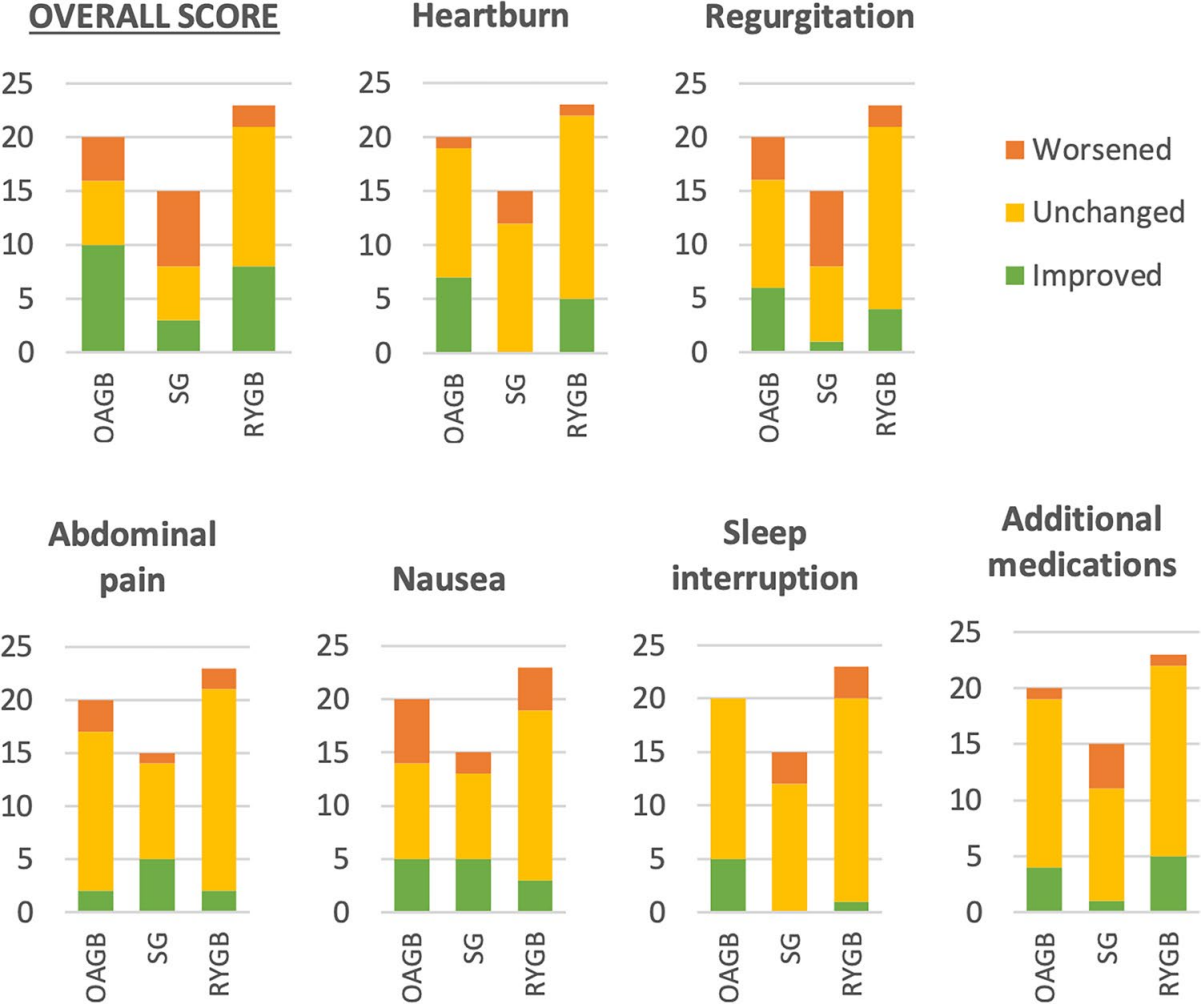
Results from GerdQ questionnaire, by category

The OAGB was the only procedure showing a statistically significant decrease in GerdQ score post-operatively (Fig. 2; Table 1). Post-operative proton pump inhibitor (PPI) usage varied among patient groups. Overall, more participants were using PPI therapy post-operatively compared with pre-operatively in all operative groups. Some participants ceased PPI medication post-operatively (OAGB, 2; SG, 0; RYGB, 2), while others were newly prescribed PPI therapy (OAGB, 5; SG, 3; RYGB, 4).

**Table 1:** Pre- and post-operative results for biometric measurements and comorbidity resolution. Results presented as mean (SD) where applicable

**OAGB** (*n* = 20)	**Pre-op**	**Post-op**	***p-value***
*Biometrics*			
BMI (kg/m^2^)	45.7 (6.9)	32.4 (4.7)	** < 0.0001**
%EWL		67.5 (18.3)	
*Comorbidities*			
Diabetes mellitus	5	1	**0.03**
HbA1c (%)	5.86 (1.1)	5.2 (0.4)	** < 0.0001**
Fasting glucose (mmol/L)	5.63 (1.5)	4.37 (0.6)	** < 0.001**
Insulin (*n* =)	0	0	-
Oral medication (*n* =)	5	1	**0.03**
Dyslipidaemia			
Total cholesterol (mmol/L)	4.63 (1.0)	4.34 (1.0)	0.29
Lipid-lowering therapy (*n* =)	5	1	**0.03**
Hypertension			
Antihypertensives (*n* =)	6	5	0.56
*Reflux symptoms*			
GerdQ score	7.6 (3.1)	6.4 (2.5)	**0.02**
PPI therapy (*n* =)	5	8	0.24
**SG** (*n* = 15)	**Pre-op**	**Post-op**	***p-value***
*Biometrics*			
BMI (kg/m^2^)	45.1 (6.1)	34.6 (6.2)	** < 0.0001**
%EWL		55.6 (18.5)	
*Comorbidities*			
Diabetes mellitus	1	1	-
HbA1c (%)	5.48 (0.4)	5.12 (0.3)	0.078
Fasting glucose (mmol/L)	5.18 (0.9)	4.1 (0.6)	**0.006**
Insulin (*n* =)	1	0	-
Oral medication (*n* =)	0	1	-
Dyslipidaemia			
Total cholesterol (mmol/L)	5.60 (1.9)	5.57 (3.7)	0.93
Lipid-lowering therapy (*n* =)	2	3	0.31
Hypertension			
Antihypertensives (*n* =)	5	3	0.13
*Reflux symptoms*			
GerdQ score	5.9 (3.5)	6.9 (2.9)	0.09
PPI therapy (*n* =)	0	3	0.05
**RYGB (***n* = 23)	**Pre-op**	**Post-op**	***p-value***
*Biometrics*			
BMI (kg/m^2^)	43.8 (6.3)	32.0 (5.6)	** < 0.001**
%EWL		68.4 (25.4)	
*Comorbidities*			
Diabetes mellitus	6	3	0.06
HbA1c (%)	5.78 (0.8)	5.28 (0.4)	**0.001**
Fasting glucose (mmol/L)	5.45 (1.3)	4.56 (0.5)	**0.003**
Insulin (*n* =)	0	0	-
Oral medication (*n* =)	6	3	0.06
Dyslipidaemia			
Total cholesterol (mmol/L)	4.36 (1.0)	3.94 (0.7)	0.10
Lipid-lowering therapy (*n* =)	6	4	0.14
Hypertension			
Antihypertensives (*n* =)	7	3	**0.03**
*Reflux symptoms*			
GerdQ score	5.9 (2.9)	6.7 (2.3)	0.08
PPI therapy (*n* =)	7	9	0.41

Bold text denotes statistically significant results with *p*-value < 0.05

*BMI*, body mass index; *%EWL*, percentage of excess weight loss; *PPI*, proton pump inhibitor; *GerdQ*, self-reported reflux questionnaire [5]

### Endoscopy

Fifty-six participants (56/58; 97%) underwent UGIE, with findings summarised in Table 2. De novo gastritis or erosion/ulceration was observed macroscopically in nine participants and histologically in six participants. Sometimes, macroscopic gastritis was not supported by microscopic findings and vice versa. Six participants (OAGB, 2; SG, 1; RYGB, 3) had macroscopic gastritis in the absence of microscopic findings, whereas three (OAGB, 1; SG, 2) participants had converse findings. De novo microscopic esophagitis was identified in eleven participants (OAGB, 5; SG, 4; RYGB, 2); however, this was only macroscopically evident in 2 participants (SG, 1; RYGB, 1). One participant, post-SG, had de novo intestinal metaplasia on distal esophageal biopsies; however, the absence of macroscopic changes suggests a sampling error. Early histological features of gastritis (foveolar hyperplasia) were observed in one OAGB patient and esophagitis (basal cell hyperplasia) in nine participants (OAGB, 5; SG, 3; RYGB, 1).

**Table 2:** Results from upper gastrointestinal endoscopy and histopathological assessment

Operation type	Pre-op	Post-op	De novo post-op	p-value*
**OAGB**				
*N* =	20 (100%)	19 (95%)		
Normal	6 (30%)	4 (21%)		0.72
Stomach				
Macroscopic gastritis	2 (10%)	1 (5%)	1 (5%)	-
Macroscopic erosion/ulceration	1 (5%)	3 (16%)	3 (16%)	0.34
Foveolar hyperplasia	0	1 (5%)	1 (5%)	0.07
*H. pylori* positive gastritis	4 (20%)	0	0	0.11
Histological acute inflammation	0	3 (16%)	3 (16%)	0.11
Histological chronic inflammation	8 (40%)	0	0	**0.003**
Oesophagus				
LA grade A esophagitis	3 (15%)	0	0	0.23
LA grade B esophagitis	1 (5%)	0	0	-
Basal cell hyperplasia	0	5 (26%)	5 (26%)	**0.02**
Histological esophagitis	5 (25%)	5 (26%)	5 (26%)	-
Intestinal metaplasia	0	0	0	-
**SG**				
*N* =	15 (100%)	14 (93%)		
Normal	7 (47%)	2 (14%)		0.11
Stomach				
Macroscopic gastritis	2 (13%)	2 (14%)	2 (14%)	-
Macroscopic erosion/ulceration	1 (7%)	0	0	-
Foveolar hyperplasia	0	0	0	-
*H. pylori* positive gastritis	0	1 (7%)	1 (7%)	0.48
Histological acute inflammation	2 (13%)	5 (36%)	3 (21%)	0.21
Histological chronic inflammation	6 (40%)	0	0	**0.02**
Oesophagus				
LA grade A esophagitis	1 (7%)	0	0	-
LA grade B esophagitis	0	1 (7%)	1 (7%)	0.48
Basal cell hyperplasia	0	3 (21%)	3 (21%)	0.10
Histological esophagitis	1 (7%)	4 (28%)	4 (28%)	0.17
Intestinal metaplasia	0	1 (7%)	1 (7%)	0.48
**RYGB**				
*N* =	23 (100%)	23 (100%)		
Normal	4 (17%)	9 (39%)		0.19
Stomach				
Macroscopic gastritis	0	1 (4%)	1 (4%)	-
Macroscopic erosion/ulceration	0	2 (9%)	2 (9%)	0.49
Foveolar hyperplasia	0	0	0	-
*H. pylori* positive gastritis	2 (9%)	0	0	0.49
Histological acute inflammation	1 (4%)	0	0	-
Histological chronic inflammation	13 (57%)	0	0	** < 0.001**
Oesophagus				
LA grade A esophagitis	3 (13%)	1 (4%)	1 (4%)	0.61
LA grade B esophagitis	1 (4%)	0	0	-
Basal cell hyperplasia	0	1 (4%)	1 (4%)	-
Histological esophagitis	4 (17%)	4 (17%)	2 (9%)	-
Intestinal metaplasia	1 (4%)	0	0	-

^*^p-value calculated using Fisher’s exact test, significant values (*p*-value < 0.05) in bold

Overall, de novo macro- or microscopic gastroesophagitis was seen in eleven participants post-OAGB (58%), eight post-SG (57%) and five post-RYGB (22%). Conversely, resolution of macroscopic esophagitis was observed in all patients post-OAGB with no de novo development of macroscopic findings. There was no statistical association between de novo gastroesophagitis and positive scintigraphy or worsened reflux symptoms.

### Gastric Fluid Analysis

Twenty-eight participants had pre-operative gastric fluid analysis for bilirubin (OAGB, 11; SG, 4; RYGB, 13), and 35 had post-operative analysis (OAGB, 14; SG, 7; RYGB, 14). No pre-operative samples measured elevated bilirubin (reference range 2–24 μmol/L). Post-operatively, elevated bilirubin levels were measured for three participants (157–498 μmol/L), all post-OAGB. No significant association was observed between elevated gastric fluid bilirubin and worsened symptoms, gastroesophagitis or positive scintigraphy.

### Biliary Scintigraphy

**Fig. 3:**
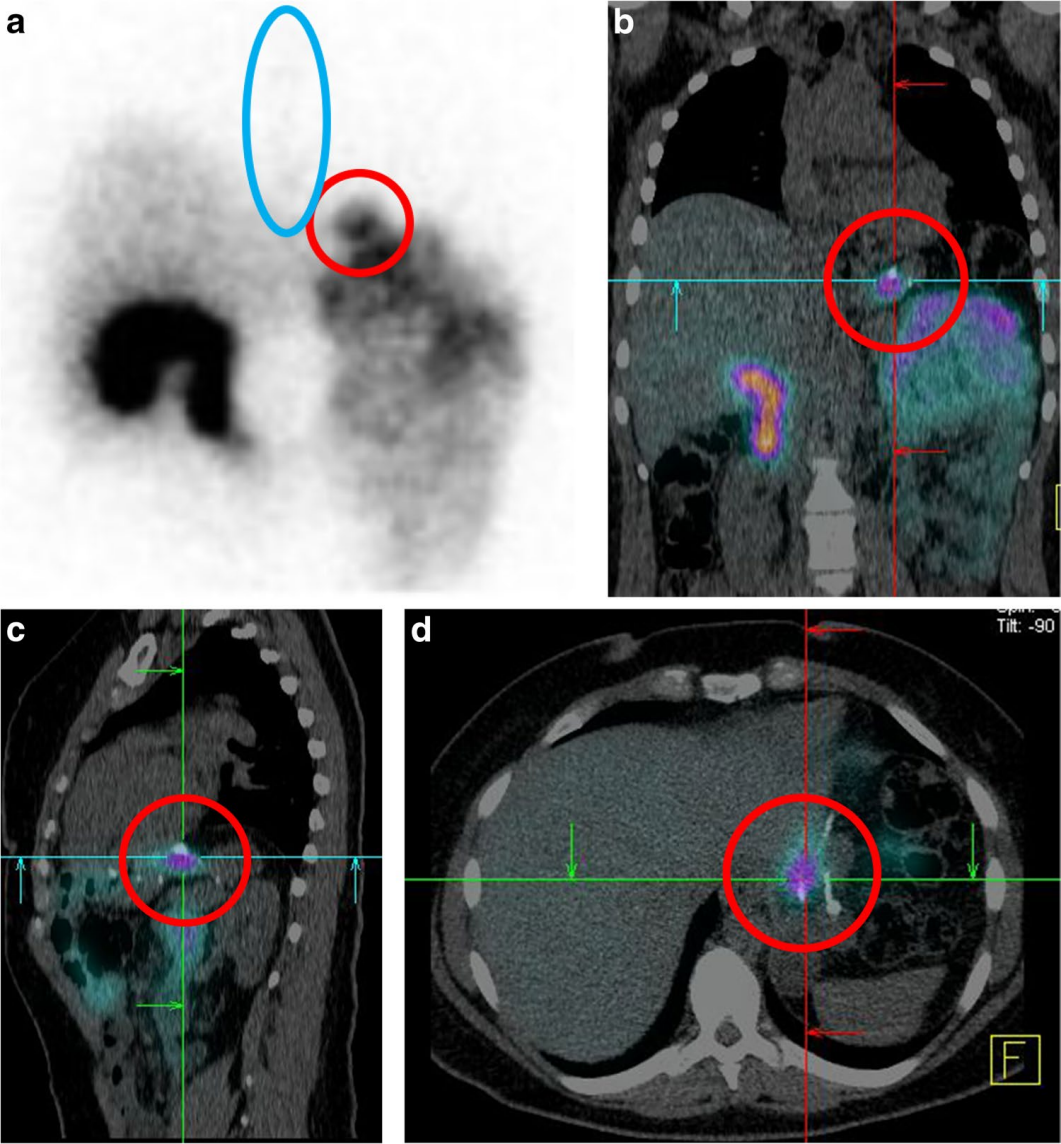
**a** Scintigraphic image showing reflux into gastric pouch (red circle) and oesophagus (blue circle). **b** and **d**: coronal (**b**), sagittal (**c**) and axial (**d**)-fused CT images, confirming localisation of reflux in the gastric pouch (red circle)

Fifty-three participants underwent scintigraphy (91%), with reflux of bile into the gastric pouch/sleeve most frequently identified in OAGB participants (70%), and to a lesser extent post-SG (31%) (Table 3). Esophageal reflux of bile was demonstrated in only one participant post-OAGB (Fig. 3). Mean percentage of reflux activity within the gastric pouch/sleeve in positive studies was low for all surgical techniques. Participants who underwent OAGB had higher likelihood of positive duodenogastric reflux on scintigraphy compared with SG (odds ratio, 1.48, 95% *CI*: 1.00–2.20, *p* = 0.05) and RYGB (odds ratio, 44.33, 95% *CI*: 2.93–670.32, *p* = 0.01). There was no statistical association between duodenogastric reflux seen on biliary scintigraphy and de novo gastroesophagitis or worsened reflux symptoms.

**Table 3:** Biliary scintigraphy results

No. scanned/total each group	OAGB	SG	RYGB
	20/20	13/15	20/23
Reflux into pouch/sleeve	14 (70%)	4 (31%)	1 (5%)
Reflux into oesophagus	1 (5%)	0	0
Reflux into gastric remnant	0	n/a	4 (17%)
Mean % reflux activity in pouch	2.9 (1.5)	4.1 (1.3)	2.05

### Weight Loss and Comorbidity Improvement

All operative groups demonstrated significant weight loss, with the greatest mean percentage excess weight loss and decrease in BMI observed for OAGB and RYGB, with less for SG (Table 1). Post-operatively, greatest improvement in glycaemic control occurred after OAGB, with significant decreases in mean HbA1c, fasting glucose, and diabetic medication usage. The RYGB and SG groups also showed improvements in glycaemic control, albeit to a lesser degree and varying statistical significance (Table 1). Improvements of hypercholesterolaemia and hypertension were observed for OAGB and RYGB, again with varying statistical significance (Table 1). No statistical significance between groups was observed for weight loss and comorbidity improvement; however, the study was not powered for such comparison.

## Discussion

This prospective study revealed that low-volume reflux of bile into the gastric pouch/sleeve is common after OAGB and SG, when compared with RYGB. The presence of duodenogastric reflux on scintigraphy had no association with reflux symptoms, nor mucosal damage of the stomach and oesophagus. In contrast, esophageal bile reflux is rare, only occurring in a single patient post-OAGB.

Diagnosing bile reflux is challenging, with no single investigation deemed superior in a systematised review of available diagnostic techniques [9]. Macro- and microscopic findings of gastroesophagitis lack specificity and thus require adjunctive investigations to confirm bile as the inciting factor. The *Sydney System* of histological assessment can aid determination of bile-related gastritis [10]. This system cannot be used in patients who have undergone gastric bypass, due to requirement of a gastric antrum biopsy. Obtaining gastric fluid for bilirubin analysis is similarly limited, due to the intermittent nature of bile reflux and dilutional impact of swallowed secretions. Biliary scintigraphy, with modifications tailored to the anatomical and physiological changes after MOS, becomes a specific and well-tolerated investigation with good sensitivity and reproducibility [8]. Combining scintigraphy for diagnosis of bile reflux, with UGIE for macro- and microscopic mucosal assessment, should now be considered the gold standard for investigation [9].

Esophageal bile reflux after OAGB has been investigated previously in five studies [11–16]. Diagnostic techniques varied, with Saarinen et al. being the only other group using biliary scintigraphy [12]. Their protocol was similar to the current study, incorporating SPECT-CT and delayed static images but omitted any provocation agents. In their two published studies, they report duodenogastric reflux incidence of 55.5% and 31.6%, lower than in this current study. Differing scintigraphy protocols may account for this discrepancy; adding a provocation agent more accurately emulates dietary intake to stimulate gallbladder emptying, improving test sensitivity [17]. Esophageal bile reflux was rarely observed in the current study, consistent with Saarinen’s results; one patient in each study demonstrated low-volume reflux of bile into the oesophagus. Low extent/severity of total reflux activity was reported in both studies, with Saarinen reporting mean activity 5.2% compared with 2.9% in the current study.

Gastroesophageal reflux (GERD) after SG has been widely reported. A recent meta-analysis of over 10,000 patients found worsened reflux symptoms post-SG in 19% of patients, de novo symptoms in 23% and long-term Barrett’s oesophagus prevalence of 8% [18]. While this review failed to elaborate on prevalence of gastric and esophageal bile reflux, a more recent study by Braghetto et al. assessed this using biliary scintigraphy in patients with de novo reflux symptoms post-SG [19]. Duodenogastric reflux was detected in 32% of patients (7/22), consistent with our results of 31% (4/13). Their scintigraphy protocol utilised an oral provocation agent but did not incorporate SPECT-CT for anatomical localisation; anatomical alterations post-SG cause less interference for scan interpretation compared with bypass procedures. Even though selection bias was evident (only symptomatic patients were enrolled), their study was like ours, in that no statistical association between symptoms and positive reflux on scintigraphy was found. Roux-en-Y gastric bypass is lauded for ameliorating GERD post-operatively [20], mechanistically attributable to hastened gastric emptying through an unrestricted gastrojejunostomy [21]. Similarly, OAGB reduces GERD post-operatively, with reduced acid exposure time, and lower number of acidic reflux events on impedance pH demonstrated 12 months post-operatively [22, 23].

The significance of demonstrated gastric and esophageal bile reflux is the potential for associated tissue damage rather than simply the presence, frequency or severity. Duodenogastric reflux can occur physiologically and is of minimal concern [24], whereas esophageal bile has the potential to be associated with mucosal damage. Esophageal mucosa exposure to bile acids increases epithelial permeability and promotes intracellular translocation of bile acids [25]. Once intracellular, bile acids can incite an inflammatory response, causing oxidative DNA damage and cell death, thus potentially initiating an esophagitis-Barrett’s-adenocarcinoma sequence [26]. The current study demonstrated de novo gastroesophagitis in 58% (*n* = 11/19) of participants post-OAGB, compared with 39.5% reported by Saarinen et al. [12]. Interestingly, the current study showed resolution of macroscopic esophagitis in all patients post-OAGB with no de novo development of macroscopic findings. This is consistent with a recent randomised controlled trial demonstrating complete endoscopic regression of pre-operative Los Angeles grade A or B esophagitis in 90% of patients at 12-month post-OAGB [23]. The long, narrow gastric pouch and widely patent gastrojejunostomy proposed as likely mechanistic explanations [22]. Studies by Lasheen and Shenouda reported post-operative gastroesophagitis in 32.5% of patients at 9 months and 55% at 6 months, respectively; however, no comment was made about whether these findings were de novo post-operative findings or had been present pre-operatively [14, 15]. Our study showed no association between de novo gastroesophagitis and positive reflux on scintigraphy, contrasting with a positive association reported by Saarinen’s group [12]. This may represent a type 2 error given the relatively small sample size.

Despite known histopathological links between esophageal mucosa exposure to bile and carcinogenesis, in the 20 years since OAGB conception, only two cases of gastric pouch/distal esophageal malignancy have been reported [27, 28]; both were adenocarcinomas of the distal oesophagus/gastroesophageal junction, diagnosed 2 years post-operatively. One patient had known Los Angeles grade C esophagitis pre-operatively; the other had no pre-operative UGIE performed. Linking post-operative bile reflux with carcinogenesis is confounded in these cases by the unclear pre-operative presence of malignancy. Further to this, a recent study compared esophageal histological changes 30 weeks after OAGB, loop esophagojejunostomy or sham operation in rats [29]. The authors demonstrated no development of precancerous or cancerous gastroesophageal lesions after OAGB, compared with the development of intestinal metaplasia in 42% of patients post-esophagojejunostomy. Similarly, based on long-term evidence from studies of patients having a Billroth II procedure, concerns of bile-related carcinogenesis may be unfounded. In a population-based study of over 18,000 patients, the incidence of gastric stump cancer post-Billroth II was no different than the expected incidence in the standard population (73/8735 cases post-Billroth II versus 83 predicted) [30]. These studies show that the actual rate of malignancy is almost certainly incredibly low, with no demonstrable link to bile reflux. Were this to be true, it would raise concern regarding RYGB, as the gastric remnant remains exposed to bile reflux from the duodenum but can never be easily visualised again. This was clearly shown in our study with 17% of RYGB patients having gastric remnant bile reflux on scintigraphy.

The number of SG procedures being performed eclipses OAGB by more than 6 times [1]. With the current study demonstrating equivalent rates of de novo gastroesophagitis between SG and OAGB, irrespective of the presence of bile, the concern surrounding carcinogenesis after OAGB must therefore be extrapolated to SG. The high prevalence of Barrett’s oesophagus after SG [18] is alarming in the context of a procedure in which a large portion of stomach is resected and discarded: the optimal surgical conduit for esophagectomy should cancer arise. Regardless of the procedure, therefore, emphasis should be placed on post-operative endoscopic surveillance, enabling early detection and treatment of Barrett, an opinion shared by the International Federation for the Surgery of Obesity and Metabolic Disorders [31].

There are limitations in this study. Participants were not randomised to their procedure, resulting in inherent selection bias; participants with pre-existing reflux or Barrett’s oesophagus on pre-operative endoscopy were positively selected for RYGB to minimise post-operative reflux [20]. The relatively small sample size will increase the likelihood of type 2 error. Equally, below-target sample size could result in an underpowered study; however, the difference in bile reflux incidence between groups was underestimated in the power calculation, ameliorating this sample size difference. Although no association between frequency of duodenogastric reflux and gastroesophagitis was demonstrated in this study, the short follow-up period may underestimate the impact bile has on gastroesophageal mucosal integrity over the long term. Finally, due to COVID-19-related government-placed restrictions, all non-essential medical treatments were ceased for 4 months during our recruitment/follow-up period. As a result, recruitment was halted, follow-up was delayed and patients withdrew due to concerns about visiting hospitals during the pandemic.

## Conclusion

In this study, duodenogastric reflux occurred frequently after OAGB but was of low volume and not associated with worsened reflux symptoms. The rarity of esophageal bile reflux, combined with post-operative resolution of macroscopic reflux esophagitis in 100% of cases post-OAGB, supports the assertion of OAGB as a safe procedure with regard to esophageal bile reflux and thus carcinogenic potential. The short follow-up period of this study may limit evaluation of the impact of chronic low-volume duodenogastric reflux on gastric mucosa. This quandary necessitates a recommendation for further medium- and long-term evaluation in this cohort of patients. However, with only two cases of malignancy post-OAGB reported in 20 years, both with no correlation to a bile aetiology, it is likely that the theoretical carcinogenic risk remains exactly that theoretical.

## Abbreviations

BMI: Body mass index
BP: Biliopancreatic
CT: Computed tomography
DGER: Duodenogastroesophageal reflux
EWL: Excess weight loss
GERD: Gastroesophageal reflux disease
HIDA: Hepatobiliary iminodiacetic acid
IV: Intravenous
MOS: Metabolic and obesity surgery
OAGB: One anastomosis gastric bypass
PPI: Proton-pump inhibitor
ROI: Region of interest
RYGB: Roux-en-Y gastric bypass
SG: Sleeve gastrectomy
SPECT: Single photon emission computed tomography
UGIE: Upper gastrointestinal endoscopy
